# Determination of Lymphocyte Cytokinesis-Block Micronucleus Values in Apparently Healthy Children by means of Age and Sex

**DOI:** 10.1155/2019/8729561

**Published:** 2019-12-25

**Authors:** Burak Durmaz, Hasan Taslidere, Guldane Koturoglu, Cumhur Gunduz, Mehmet Orman, Ozgur Cogulu

**Affiliations:** ^1^Department of Medical Genetics, Ege University, Faculty of Medicine, Izmir, Turkey; ^2^Department of Pediatrics, Ege University, Faculty of Medicine, Izmir, Turkey; ^3^Department of Medical Biology, Ege University, Faculty of Medicine, Izmir, Turkey; ^4^Department of Biostatistics, Ege University, Faculty of Medicine, Izmir, Turkey

## Abstract

The cytokinesis-block micronucleus (MN) assay on blood lymphocytes is one of the most important tests implemented in cytogenetics for the measurement of genotoxicity. For the purpose of biological dosing, it is crucial to know the spontaneous frequency of MN and its normal values in general population, especially in children, which are used for the population databases. In this study, MN levels were investigated in cytokinesis-blocked lymphocytes of 150 apparently healthy children aged 1 to 15. Our aim was to assess the variability of MN values according to age and sex. The mean MN frequency among boys was 3.69 ± 1.747‰ and 4.12 ± 1.867‰ in girls where there was no significant difference in relation to age and sex. However, when we separated age groups as 0–2 years, 3–5 years, 6–10 years, and 11–15 years, one-way ANOVA test showed significant association. Significance was obvious in the 0–2 years age group with the 3–5 years age group and 6–10 years age group. When we grouped our study population as 0–2 years and 3–15 years, the mean MN frequency among the 0–2 years age group was 2.85 ± 1.599‰ and 4.07 ± 1.867‰ in the 3–15 years age group which was also statistically significant. This difference may be attributed to age-related increase of close contact with environmental hazardous agents. In conclusion, normal values of MN obtained in this study will add valuable information in regard to update the current childhood population data and will act as a reference for further genotoxicity studies.

## 1. Introduction

Maintaining the normal function of a cell is crucial for a living organism, and it depends largely on proper DNA replication and repair mechanisms. In our daily lives, we are increasingly exposed to genotoxic effects including ionizing radiation, detrimental chemicals, and harmful physical agents. These genotoxic agents gradually damage the genetic information within a cell and may predispose to cancer, chronic diseases, or apoptosis [1, 2]. The effect of those factors on genomic stability could be determined by the measurement of micronuclei (MN) [3]. MN which originates from chromosome fragments or whole chromosomes is a measure of chromosome breakage or loss. The most commonly applied method for assessing genotoxicity by MN assay is the cytokinesis-block technique in which cytochalasin-B is used for this purpose [4]. Elevated MN frequency in lymphocytes has been shown to be associated with an increased risk of cancer, severe adverse cardiovascular events in coronary artery disease patients, diabetes, mild cognitive impairment, Alzheimer's, and Parkinson's diseases [5–8]. Most of the studies investigating MN have been conducted in adults. In parallel, the great majority of studies performed in children has been on the effects of environmental factors, although there have been some reports of increased MN values among children with malnutrition, obesity, iron deficieny, and low blood levels of vitamin B12 [9–12]. Although this test has been reported to be reliable and useful, large variations are observed in the literature [13, 14]. This variability has been linked to study designs, different experimental conditions, and population characteristics such as genetic and lifestyle factors [15]. In the light of those observations and reports, requirement of normal reference values has been pointed out by some authors [14, 15]. In this study we aimed to present the age-specific MN values in 150 apparently healthy children aged 1 to 15.

## 2. Materials and Methods

### 2.1. Patient Selection

Local Ethics Committee approval of the Institute of Medical Research was obtained before starting this work, and this study was performed in accordance with the ethical standards laid down in the Declaration of Helsinki. All the patients and parents were properly informed before and during the procedure, and they signed the informed content. The study population consisted of 150 apparently healthy children who were referred to pediatric outpatient clinic for routine follow-up. The selected children were all selected from the same locality in the summer period (June-August) where there is very limited air pollution and infections. The families were questioned about smoking habits, occurrence of cancer and malignancies, and history of vaccination, and the subjects were selected accordingly. The participants were grouped as age 1 to 15, each consisting of 10 children (5 boys and 5 girls). Inclusion criteria for the study comprised that all the subjects were healthy at the time of blood sampling, none of them had been exposed to ionizing or nonionizing radiation for diagnostic or other purposes during the six-month period, none of them took any medication and none of them had inherited genetic disorders or chronic diseases, and all the children were having a balanced diet and they were all within normal body mass index.

### 2.2. Blood Sampling

Peripheral whole blood samples were collected in heparinized tubes under aseptic conditions in the morning hours. After collection, the samples were randomly coded and transferred to the laboratory and processed on the same day.

### 2.3. Cytokinesis-Block Micronucleus (CBMN) Assay

**Figure 1:**
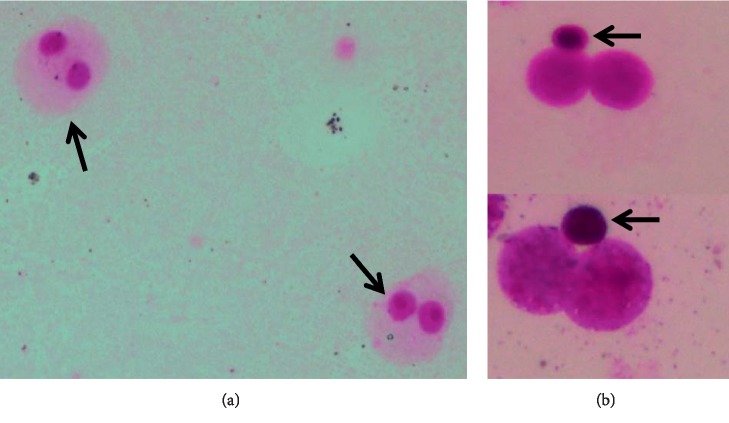
Representative of images of cells scored in the micronucleus study: (a) normal binucleated cells, shown by black arrows and (b) close-up view of binucleated cells with a micronucleus. The micronuclei are shown with black arrows.

The CBMN assay was performed using cytochalasin B (Cyt-B) as described elsewhere [2]. The cells from peripheral venous blood were cultured in the RPMI medium supplemented with fetal bovine serum, phytohaemagglutinin, penicillin, and streptomycin at 37°C for 72 hours. Cyt-B at a final concentration of 3 *μ*g/ml was added to each sample on the 44th hour of incubation to accumulate cells that had divided once and incubated for another 18 hours, and then the cultures were harvested. They were centrifuged at 1100 rpm for 10 minutes. After the supernatant was removed, 10 mL of prewarmed hypotonic solution was added to the pellet and incubated for 23 min at 37°C. The cultures were centrifuged at 1100 rpm for 10 minutes and the supernatant was removed, and cells were fixed with a fixative, methanol-acetic acid (3 : 1, v/v). The cells were resuspended in a small volume of fixative and dropped onto clean cold slides. Finally, the slides were stained for 10 minutes in 5% Giemsa dye. Every subject was analysed for the total number of micronuclei per 1000 binucleated cells according to criteria previously published by Fenech [16]. To prevent commentary differences, the cells were scored under the light microscope by two different qualified and experienced Medical Genetics specialists. Only cells with well-defined cytoplasmic border were evaluated for scoring (Figure 1). Microscopic analysis was done with an optical microscope with a final magnification of 400x (Olympus BX50, Tokyo, Japan).

### 2.4. Statistical Analysis

The statistical analysis was performed using SPSS 25.0 (SPSS Inc., IL, USA) statistical program package. The Student's *t*-test was used to compare the frequencies of MN among study groups. The Pearson method (*r*) was performed to evaluate the association between age, gender, and MN frequencies. The one-way ANOVA test was used to evaluate the association between MN frequencies and different age groups, and the Bonferroni test was conducted for multiple comparisons of age groups. The value of *p* < 0.05 was considered statistically significant.

## 3. Results

**Figure 2:**
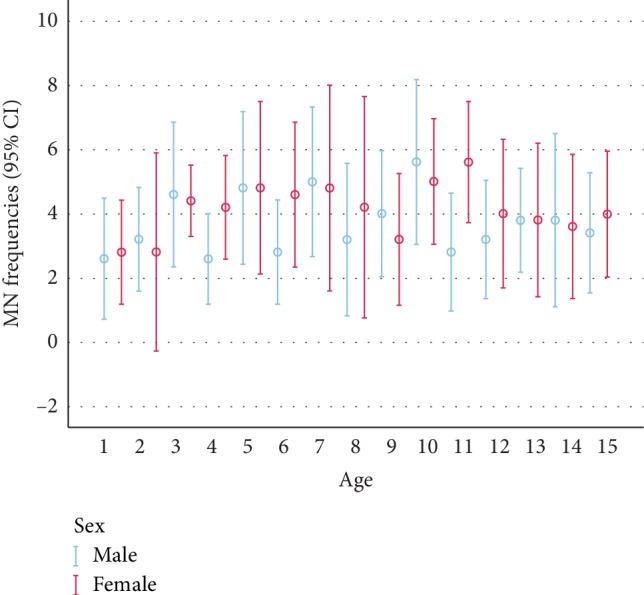
Error bar chart representing the MN frequencies, ages, and sexes of the study group. The frequency of MN dispersed evenly among sex and age (95% confidence interval) (MN: micronucleus, CI: confidence interval).

**Figure 3:**
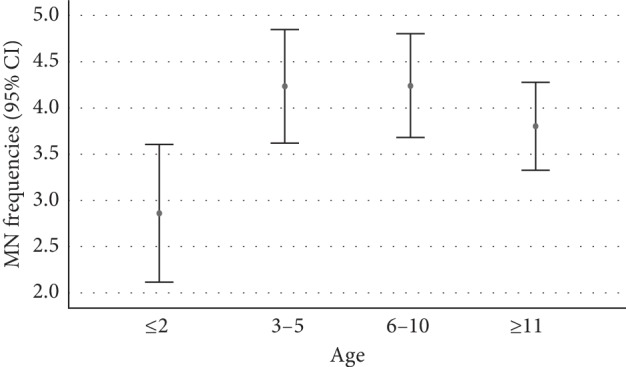
Error bar chart shows the MN frequencies between different age groups. There is a significant association of MN frequencies of the 0–2 years age group with the 3–5 years age group and 6–10 years age group (95% confidence interval) (MN: micronucleus, CI: confidence interval).

**Figure 4:**
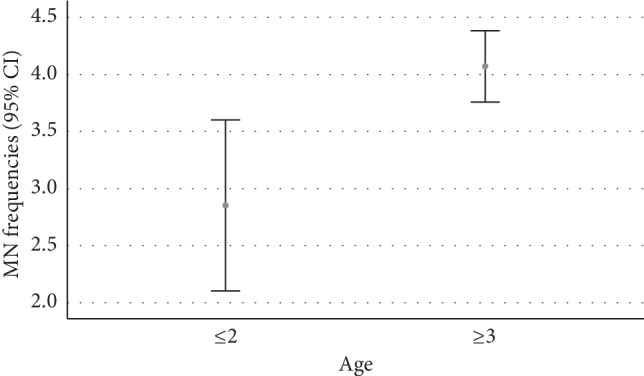
Error bar chart reveals the MN frequencies of 0–2 years age group and 3–15 years age group. There is a significant association of MN frequencies between these 2 groups (95% confidence interval) (MN: micronucleus, CI: confidence interval).

The study population consisted of 150 healthy children, 75 girls (50%), and 75 boys (50%) with ages ranging from 0 to 15 years. Each age group consisted of 10 subjects (5 boys and 5 girls).  Figure 2 presents the error bar graph of MN frequencies within this group. The distribution of MN frequencies dispersed evenly when compared with age and sex. The mean MN frequency among boys was 3.69 ± 1.747‰ and 4.12 ± 1.867‰ in girls (95% confidence interval (CI)). Student's *t*-test showed there was not any significant correlation between males and females in regard to MN results (*p*=0.151) (Table 1). The Pearson correlation revealed that there was no significant difference between males and females in relation to age (*r* = 0.073; *p*=0.372) and sex (male *r* = 0.55, *p*=0.639 and female *r* = 0.92, *p*=0.435) (Table 2). However, when we compared different age groups, we were able to find significant results. We separated age groups as 0–2 years, 3–5 years, 6–10 years, and 11–15 years, and the one-way ANOVA test revealed significant association between these groups (*p* = 0.021) (Table 3). Bonferroni posthoc test for multiple comparisons showed significant association of MN frequencies of the 0–2 years age group with 3–5 years age group (*p*=0.046) and 6–10 years age group (*p*=0.021). When we grouped our study population as 0–2 years and 3–15 years, the mean MN frequency among the 0–2 years age group was 2.85 ± 1.599‰ and 4.07 ± 1.867‰ in the 3–15 years age group (95% confidence interval (CI)). Student's *t*-test revealed significant association of MN frequencies between these groups (*p*=0.005) (Table 4). Figures 3 and 4 show the error bar graphs of MN frequencies among different age groups.

## 4. Discussion

In parallel with advances in civilization and developments in technology, the rate of exposure of human life to many genotoxic agents has been increasing over time. The foods taken, the drugs used, and the different causes of radiation are some of those leading agents which come to mind in the first place. Therefore, it is important to measure the genotoxic capacity of these agents on human health. By this way, it can be possible to screen new chemicals which may be hazardous for the environment, to determine the acceptable level of genetic damage, to identify individuals who are at risk for a specific genotoxic agent. Among the methods measuring genotoxicity in different situations, micronucleus assay is one of the most preferred methods. A number of studies were carried out to determine the baseline frequencies of MN measured with the CBMN method in standard healthy populations. The main factors to affect the frequency of MN were analysed, and they are confirming the age and sex as the main factors associated with the frequency. However, as pointed out by some studies, normal values for micronucleus test in healthy populations has been suggested by some authors due to the wide range of results of this test [14, 15, 17]. Considering this valuable information, the mean MN frequency of all participants in our study was found to be 3.91 ± 1.82‰, 3.69 ± 1.75‰ in female group, and 4.12 ± 1.87‰ in male group. Whereas those values were reported to be higher in adults such as 5.06 ± 3.11‰ in all subjects, 5.16 ± 2.98‰ in females, and 4.79 ± 3.44‰ in males by Gajski et al. [15]. The major difference between those studies and our study is the age groups of the participants. Gajski et al. included adult individuals between 19–80 years of age into their study and separated the study groups as <30, 31–40, 41–50, 51–60, 61–70, and >70. The highest value was 6.47 ± 2.42‰ in the 61–70 age group, and the lowest was in the <30 age group which was 4.15 ± 2.79‰. Considering much lower value obtained in our study, increased ratios of MN in previous similar studies could be attributed to age-dependent increasing exposure of environmental conditions which may cause genomic damages.

In our study, no significant correlation was detected between male and female groups. The mean MN frequency of the female group was higher compared with the male group. Age and sex were found to have significant influences on MN frequency by Gajski et al. [15]. Although not being significant, a higher mean value of MN in the female group in our study may be associated with many factors such as growth characteristics of two sexes. In parallel with our study, Bonassi et al. reported not significant but higher level of MN frequency in females compared with males [18].

One of the interesting finding of our study was the detection of significant difference between the 0–2-year-old age group and the other age groups except 11–15. The lowest value in all studies including our study was detected in 0–2 age group. This difference may be due to the fact that children in this age group are exposed to environmental effects much less compared with other age groups because they are in infancy. The fact that children end up in infancy and transition to early childhood brings with it many problems. Common view of the researchers is that the early childhood period is the most important development period in life. They begin to learn the world around them and become vulnerable to harmful external effects such as food or physical and chemical agents by spending more time in the outdoor environment. Therefore, this close contact with hazardous agents increases with age as it progresses to an age-related relationship. Nevertheless, no linear correlation was found with the increasing age in our study except the mean MN values between the different age groups which were identified according to the growth and human development. The fact that there is no linear significant correlation related to each age can be attributed to individual differences in each age group.

In conclusion, our study is one of the few studies discussing the age-related baseline MN frequencies in the pediatric age group. Normal values in this study will be used to update the current pediatric population data regarding micronucleus and will act as a reference for future genotoxicity studies.

## Figures and Tables

**Table 1:** Comparison of MN frequencies by means of sex. There was no significant correlation (*p* > 0.05).

Sex	Number (%)	MN mean ± SD	*p*
Male	75 (50)	3.69 ± 1.747	0.151
Female	75 (50)	4.12 ± 1.867	
Total	150 (100)		

MN: micronucleus, SD: standard deviation.

**Table 2:** The Pearson correlation revealed no significant difference between MN results, sex, and age (MN: micronucleus).

	Pearson correlation (*r*)	*p*
Sex		
Male	0.55	0.639
Female	0.92	0.435
Age	0.073	0.372

**Table 3:** The number of cases and MN frequencies are given among different age groups. The one-way ANOVA test showed significant correlation between these groups.

Age group (years)	Number (*n*)	MN Mean ± SD	*p*
0–2	20	2.85 ± 1.599	0.021^*∗*^
3–5	30	4.23 ± 1.654	
6–10	50	4.24 ± 1.985	
11–15	50	3.80 ± 1.678	
Total	150	3.91 ± 1.815	

MN: micronucleus, SD: standard deviation, ^*∗*^ = statistically significant *p* value.

**Table 4:** The Benferroni test revealed significant association of MN frequencies of the 0–2 years age group with the 3–5 years and 6–10 years age groups (upper part). Student's *t*-test revealed significant association of MN frequencies between the 0–2 years age group and 3–15 years age group (lower part).

Compared age group (years)	Age group (years)	*p*
0–2	3–5	0.046^*∗*^
	6–10	0.021^*∗*^
	11–15	0.268

0–2	3–15	0.005^*∗*^

^*∗*^ = statistically significant *p* value.

## Data Availability

The data used to support the findings of this study are included within the article.
